# A Composition Design Strategy for Refractory High-Entropy Alloys

**DOI:** 10.3390/ma18194493

**Published:** 2025-09-26

**Authors:** Faling Ren, Yilong Hu, Ruitao Qu, Feng Liu

**Affiliations:** State Key Laboratory of Solidification Processing, School of Materials Science and Engineering, Northwestern Polytechnical University, 127 West Youyi Road, Xi’an 710072, China

**Keywords:** high-entropy alloy, elastic modulus, composition design, strength, ductility

## Abstract

How to rationally design composition of alloys with desired properties has always been an open and challenging question, especially for high-entropy alloy (HEA) which has huge selections of composition due to the feature of multi-principal elements. Although great efforts have been made in the past decades, such as approaches based on thermo-kinetic analysis and simulations, strategies to quick determine the optimal HEA composition remain lacking. In this study, based on the effective estimations of elastic modulus of alloys from compositions, we proposed a strategy to design intrinsically strong, ductile, and low-weight refractory HEA (RHEA) compositions. First, the Young’s moduli of three RHEAs were experimentally measured using uniaxial tensile test and impulse excitation of vibration (IEV) test. Then, the present results, combining with the data of elastic moduli of ~130 HEAs in literature, were utilized to validate the prediction of elastic moduli from compositions of HEAs. Finally, based on the property maps that containing 38,326 compositions, a novel RHEA was designed and experimentally tested, exhibiting superior strength, ductility, and low density compared to the equimolar NbMoTaVW alloy. This study provides a new strategy for developing HEAs and contributes to the development of new refractory HEAs with desired properties.

## 1. Introduction

High-entropy alloys (HEAs) have recently received extremely high research attention owing to their very promising mechanical properties and interesting structure characteristics [1,2,3,4,5,6,7]. A well-known advantage of HEAs is their wide range of compositional space, because these alloys are often composed of multiple principal components [8,9]. There are more than three kinds of principal elements in HEAs, and HEAs are also called multiple-principal element alloys (MPEAs) [2,8]. This naturally raises a question: What is the direction for designing the HEA composition to achieve the optimal properties?

Material design for promising mechanical properties, such as high strength and high ductility, has always been a hot topic in the field of metallic structural materials, and many strategies have been proposed [10,11], e.g., the generalized stability principle based on thermodynamics and kinetics [12,13,14,15]. In recent years, great efforts and significant approaches have been made for the composition design of HEAs [16,17,18,19,20,21]. For example, researchers have designed the HfNbTiVAl_10_ alloy with a hierarchical heterostructure featuring multilevel chemical fluctuations, which exhibits an excellent combination of tensile strength and yield strength [19]. In addition, researchers have also applied various computational simulation methods for material design. For example, thermodynamic calculations based on thermophysical parameters and calculation of phase diagram (CALPHAD) [22,23], first-principle calculations combined with high-throughput calculations [24,25,26], and machine learning modeling have already discovered some alloys with excellent properties [27,28,29,30]. Despite the great progress, the large calculation amount and relatively low efficiency in simulations, as well as the difficulty in predicting the fracture property such as toughness, limit the quick discovery of new good HEAs. So, can we predict the properties of HEAs, such as Young’s modulus or yield strength, directly from their composition?

In 2012, Wang [31] proposed a prediction method for the mechanical and physical properties of metallic glasses based on their compositions, linked to the elastic modulus, providing a unique approach to designing the composition of metallic glasses from an elastic perspective. Further efforts, including the precise prediction of elastic modulus by Liu et al. [32,33] and the prediction of failure behavior by Wu et al. [34,35,36], confirm the significance of the concept of composition design for metallic glasses from elastic modulus, all of which have demonstrated high prediction accuracy. Inspired by these approaches, in this study, we investigate the elastic modulus of HEAs and attempt to examine the feasibility of the above concept of composition design from an elastic perspective for HEAs.

On the other hand, there have been numerous discussions on the correlations between elastic modulus and the mechanical properties of various materials. For instance, both the strength and hardness of materials scale with Young’s modulus, and Poisson’s ratio or Pugh’s ratio [37] (i.e., the ratio of bulk modulus B to shear modulus G) have been considered to be closely related to ductility and/or toughness. Besides the elastic criteria, there are also studies on predicting the ductility of body-centered cubic (BCC) HEAs from valence electron concentration (VEC) [38] and predicting the formation of single-phase structure from the atomic size difference [39]. The atomic size difference can be expressed by the following formula:

(1) $$\begin{array}{c} \delta =\sqrt{\sum_{i=1}^{n}c_{i}{\left(1-\frac{r_{i}}{\bar{r}}\right)}^{2}} \end{array}$$

where $c_{i}$ and $r_{i}$ represent the atomic percentage and atomic radius of the *i*-th element, respectively, and $\bar{r}=\sum_{i=1}^{n}c_{i}r_{i}$ denotes the average atomic radius. When δ < 0.066, the alloy tends to form a single-phase solid solution. These approaches are also important for the composition optimization of HEAs.

Refractory high-entropy alloys (RHEAs) exhibit excellent high-temperature strength and stability. For example, NbMoTaVW maintains a stable BCC single-phase structure at 1400 °C, with a yield strength of 477 MPa at 1600 °C [40]. However, it shows less than 5% compression fracture strain at room temperature, exhibiting significant brittle cleavage fracture behavior, which severely limits its application. Therefore, this study focuses on the NbMoTaVW alloy system, aiming to design new RHEAs that offer strength similar to NbMoTaVW but with better plasticity, lower density, and greater potential for practical applications.

In this study, we first measured the elastic modulus of three RHEAs using two experimental methods, i.e., uniaxial tension and impulse excitation of vibration (IEV). We then studied the data of elastic modulus in the literature for many alloys with different compositions, including both experimentally measured data and computer-simulated values of elastic modulus. Based on the elastic modulus data in the present study and in the literature, we examine the validity of the mixture law for predicting the elastic modulus. Finally, based on the NbMoTaVW alloy system, property maps were generated through the predictions of Young’s modulus, valence electron concentration, and density, and a new alloy composition was designed and experimentally tested. Unlike traditional methods, which often rely heavily on large-scale simulations and complex computational models, our approach offers a more direct and efficient prediction of the elastic modulus of HEAs based on their composition. This method provides a simpler framework, enabling faster and more accessible composition optimization. The novelty lies in applying this composition design concept, inspired by previous work on metallic glasses, to RHEAs, thus paving the way for the design of high-performance alloys with optimized properties.

## 2. Composition Design Strategy

The key to the concept of designing HEAs from an elasticity perspective includes two aspects: (1) the predictions of the elastic modulus from composition, and (2) the predictions of properties from the elastic modulus. Although some approaches to studying the elastic modulus of HEAs have been studied, they mainly focused on the experimental measurement of modulus, the comparison between experiments and simulation, and the factors affecting the elastic modulus. These approaches are helpful for understanding the elastic properties of HEAs; however, they do not provide direct predictions of the elastic modulus. In order to predict the elastic modulus directly from the composition, there are two methods, as discussed in the following section.

(1) The ROM method

Greer et al. [41] proposed the rule of mixtures (ROM) method to estimate the elastic modulus of an alloy from its composition. In this approach, two limiting assumptions are considered. Under the iso-strain condition (uniform strain in all constituent elements), the alloy modulus is obtained as a volume fraction weighted average of the elemental moduli, which provides the upper bound of the alloy modulus. Conversely, under the iso-stress condition (uniform stress across all constituents), the alloy modulus is calculated as the harmonic mean of the elemental moduli, which gives the lower bound. Mathematically, these can be expressed as

(2) $$\begin{array}{c} P=\sum \frac{P^{i}V_{m}^{i}f^{i}}{V_{m}}\;\left(iso-strain\right) \end{array}$$

(3) $$\begin{array}{c} \frac{1}{P}=\sum \frac{V_{m}^{i}f^{i}}{{P^{i}V}_{m}}\;\left(iso-stress\right) \end{array}$$

where P is the elastic modulus of alloy (could be *E*, *G,* or *B*); $P^{i}$ and $V_{m}^{i}$ are the modulus and molar volume of the *i*-th element, respectively; $f^{i}$ is its atomic fraction; and $V_{m}$ is the average molar volume of alloy, given by $V_{m}=\sum V_{m}^{i}f^{i}$. The values predicted by these two assumptions, therefore, represent the theoretical bounds of the alloy modulus. For metallic glasses, the ROM method has been proven to be a useful method for predicting the elastic modulus [31,32], while the applicability of this method for HEAs remains unknown.

(2) The Johnson method

Recently, Johnson et al. [42] found direct correlations between elastic moduli, strengths, and properties of the electron gas for various alloys, and hence proposed a method to quickly estimate the modulus and strength of HEA. The relation between the bulk modulus *B* and *r*_s_, the local exchange correlation parameter related to the interstitial electron density $\rho_{0}$ (i.e., $\frac{4}{3}\pi r_{s}^{3}\equiv \frac{1}{\rho_{0}}$), can be written as follows [42]:

(4) $$\begin{array}{c} B=2403.4r_{s}^{-4.546} \end{array}$$

Johnson et al. [42] also calculated the *r*_s_ of all metal elements in the periodic table, and thus the *r*_s_ of the alloy was obtained by the rule-of-mixture. They further assumed Poisson’s ratio *ν* = 1/3; thus, Young’s modulus, *E* = 3(1 − 2*v*)*B* = *B*, can be estimated.

The applicability of the two Young’s modulus calculation methods to HEAs is discussed in detail in Section 4.2. The problem of predicting the elastic modulus from the composition is solved, and there is a further need to predict the properties from the elastic modulus. We note that the ideal tensile strength scales with the Young’s modulus for various materials [43,44], i.e.,

(5) $$\begin{array}{c} \sigma_{th}=\alpha E \end{array}$$

here, *α* is a material constant. Hence, materials with a higher Young’s modulus usually tend to intrinsically possess greater strength. Therefore, in this paper, alloy compositions with high Young’s modulus are selected, which are considered to have high strength.

On the other hand, regarding the ductility of materials, there are also some relations for prediction [37]. For HEAs, Qi et al. [45] found that reducing the valence electron concentration on the one hand causes the Fermi energy level to move down relative to the band structure, resulting in earlier shear instability of the material. On the other hand, it is also possible to increase the density of states (DOS) near the Fermi level, thereby increasing the driving force of the Jahn–Teller distortion. After the Jahn–Teller distortion, the DOS peaks slightly below and above the Fermi level move further away from the Fermi level to reduce energy. In this way, the alloy exhibits intrinsic ductility. In addition, Chan proposed [46] that reducing the *VEC* in the Nb-based alloy can reduce the Peierls–Nabarro energy barrier, thereby improving the dislocation mobility and improving the ductility of the alloy. Sheikh et al. [38] proved that the criterion based on the *VEC* could predict the intrinsic ductility, especially for the refractory HEAs. Given the alloy composition, the *VEC* of the alloy can be obtained according to

(6) $$\begin{array}{c} VEC=\sum f_{i}{VEC}_{i} \end{array}$$

where $f_{i}$ and *VECi* denote the atomic percentage and the *VEC* of the *i*_th_ elements, respectively. Recently, Mak et al. [47] proposed that the *VEC* criterion for intrinsic ductility indicates that materials are brittle when *VEC* > 5 and ductile when *VEC* ≤ 5. Thus, HEAs with low *VEC* values can be selected and expected to show good plasticity. However, high-VEC HEAs, such as NbMoTaVW (*VEC* = 5.4), exhibit low fracture strain (less than 5%) [40], further supporting the notion that high VEC values tend to limit ductility.

Moreover, it has been proven that the density of HEAs can be predicted well according to the following equation [48]:

(7) $$\begin{array}{c} \rho =\sum c_{i}A_{i}/\sum \left(\frac{c_{i}A_{i}}{\rho_{i}}\right) \end{array}$$

where $c_{i}$, $A_{i}$, and $\rho_{i}$ denote the atomic concentration, mass, and density of the *i*_th_ elements, respectively.

**Figure 1:**
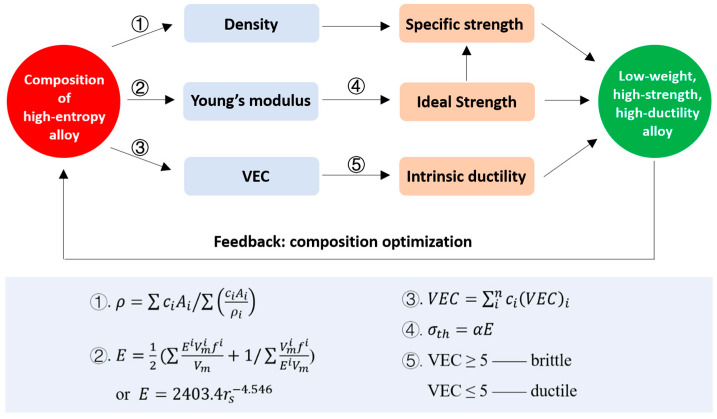
Illustration of a design strategy for composition of HEAs.

Equations (2)–(7) present the correlations between the HEA composition and properties. Based on these equations, one could expect an approach to design HEA compositions with excellent properties, such as high modulus (high intrinsic strength), high potential ductility, and low weight, as illustrated in Figure 1. To sum up, the composition design strategy includes three steps:

First, select the composition system. We chose the Nb-Mo-Ta-V-W system as an example. The atomic concentrations of the elements are then varied step by step to change the composition systematically.

Second, the elastic modulus, *VEC*, and density are calculated according to the relevant equations for each composition.

Third, the predicted property maps, such as plots of *E* vs. *VEC* and density vs. *E*, can be drawn, and the specific material composition with the required property can be obtained from the maps. For instance, the HEA with the composition of low *VEC* and high *E* is expected to show intrinsically high strength and high ductility, while the alloy composition with high specific modulus can be found in the property map of density vs. *E*.

## 3. Experimental Methods

Three refractory HEA ingots with compositions of Al_0.5_Mo_0.5_NbTa_0.5_TiZr, NbTaTiV, and HfNbTaTiZr were prepared via vacuum levitation melting (VLM) and casting. During VLM, the molten liquid alloy was electromagnetically stirred, which enabled the melts to be fully mixed and led to a uniform and accurate composition. Moreover, the alloy was suspended in the crucible in the VLM process; thus, a high-purity alloy can be prepared without contamination. The ingots have dimensions of Ø50 mm × 50 mm, which are large enough for the subsequent measurements of tensile properties and elastic modulus.

**Figure 2:**
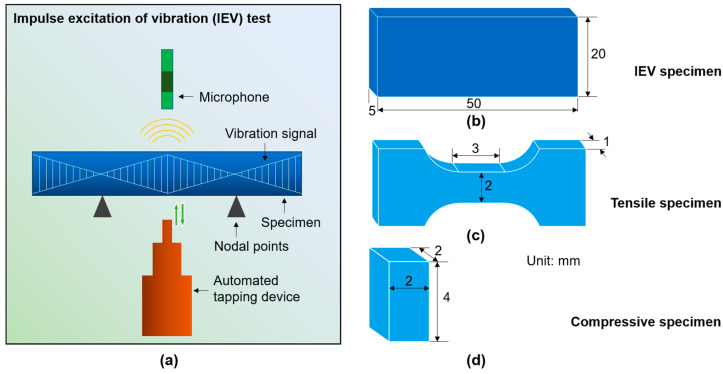
(**a**) Sketch of experimental set-up of IEV for testing the elastic moduli. (**b**–**d**) Shapes and dimensions of the IEV, tensile, and compression specimens, respectively.

Tensile specimens in the shape of a dog bone with gauge dimensions of 3 × 1.7 × 1 mm^3^ were processed from NbTaTiV and HfNbTaTiZr alloy ingots by an electric spark cutting machine. Figure 2c shows the dimensions and shape of the specimen for tensile testing. Before testing, all specimens were ground and polished to remove surface impurities. Uniaxial tensile testing was performed at room temperature using an Instron 5967 testing machine with a strain rate of 5 × 10^−4^ s^−1^. To ensure the reliability and repeatability of the results, at least three specimens were tested for each alloy under the same experimental conditions. A video-based extensometer was used to record the deformation features on the specimen surfaces and to measure the strains. During the tensile tests, the applied load and elongation were continuously recorded. These data were converted into engineering stress–strain curves, from which the yield strength, ultimate tensile strength, and total elongation were obtained. The elastic modulus was determined from the linear portion of the stress–strain curve according to Hooke’s law. For the Al_0.5_Mo_0.5_NbTa_0.5_TiZr HEA, no valid tensile data were obtained because the material was too brittle to be tested correctly.

Moreover, the IEV test was also carried out to measure the elastic moduli for the three HEAs. A sketch of the IEV test setup is shown in Figure 2a. In the IEV tests, the recorded quantities included the flexural and torsional resonance frequencies, together with the specimen mass and dimensions. The IEV test measures the elastic properties based on the measurement of the mechanical resonance frequency of a specimen with regular geometry, because the specific mechanical resonance frequency of the material is determined by the elastic modulus, mass, and the specimen geometry. For a rectangular bar specimen, the Young’s modulus (*E*) and shear modulus (*G*) are measured based on the flexural and torsional resonance frequencies, respectively, according to the following equations [49]:

(8) $$\begin{array}{c} E=0.9465\frac{mf_{f}^{2}}{b}\left(\frac{L^{3}}{t^{3}}\right)T_{1} \end{array}$$

(9) $$\begin{array}{c} G=\frac{4Lmf_{t}^{2}}{bt}R \end{array}$$

Here *m*, *b*, *L*, and *t* represent the mass, width, length, and thickness of the specimen, respectively; $f_{f}$ and $f_{t}$ are the flexural and torsional resonance frequencies, respectively; *T*_1_ and *R* are specimen size dependent correction factors. For Poisson’s ratio, it is calculated based on the measured Young’s modulus and shear modulus, assuming the isotropic elastic behavior of the materials, according to

(10) $$\begin{array}{c} \nu =-1+\frac{E}{2G} \end{array}$$

Rectangular bar specimens with dimensions of 50 mm × 20 mm × 5 mm were processed from the ingots of the three HEAs via wire cutting for IEV tests. Figure 2b illustrates the specimen dimension and shape for IEV testing. According to the ASTM E1876 standard, the IEV test was performed by using the IMCE NV elastic modulus analyzer (RFDA HT1600). It is important to note that Raggio et al. [48] performed IEV tests using seventeen steel specimens and found that the variation of the measured acoustic velocities is less than 1% for compressional wave velocity and 0.2% for shear wave velocity. Since the compressional and shear wave velocities were determined directly by the density and elastic moduli of materials, these results suggest that the errors (variation) for testing elastic moduli by IEV are expected to be rather limited. Owing to the large size of the IEV specimen and the limitation of the casting alloy, one IEV specimen for each HEA was tested.

Finally, for the testing of the designed new HEA, as discussed in Section 4.3, we prepared the alloy ingot using the electric arc melting method under argon protection. The phase composition of the HEA was then characterized by X-ray diffraction (XRD, Co Kα radiation, Bruker D8 DISCOVER A25 model) from 2θ = 20° to 140°. We also performed backscattered electron (BSE) imaging using scanning electron microscopy (SEM) to confirm the microstructure of the as-cast alloy. The specimens for XRD and SEM were ground using 2000-grit sandpaper and then polished with diamond paste. The SEM and BSE analyses were carried out using a ZEISS Sigma300 field emission scanning electron microscope under a voltage of 20 kV. To measure the mechanical property, uniaxial compression tests were carried out using an Instron 5967 testing machine. Before compression, the specimen surfaces were carefully ground and polished with a final specimen size of ~2 × 2 × 4 mm^3^ (as sketched in Figure 2d). The tests were performed at room temperature and a quasi-static strain rate of 5 × 10^−4^ s^−1^. At least three specimens were tested to ensure the reproducibility of the results. After compression, the surface morphologies of the deformed specimens were observed using SEM. Moreover, to further characterize the mechanical properties of the new alloy, Vickers hardness testing and nanoindentation were performed on the polished specimen surface. For the Vickers hardness test, the load for all the specimen positions was 200 g and held for 5 s. A nanoindentation test was conducted using a Bruker Hysitron TI 980 nanoindenter.

## 4. Results and Discussion

### 4.1. Elastic Modulus Experimentally Measured by Tension and IEV

**Figure 3:**
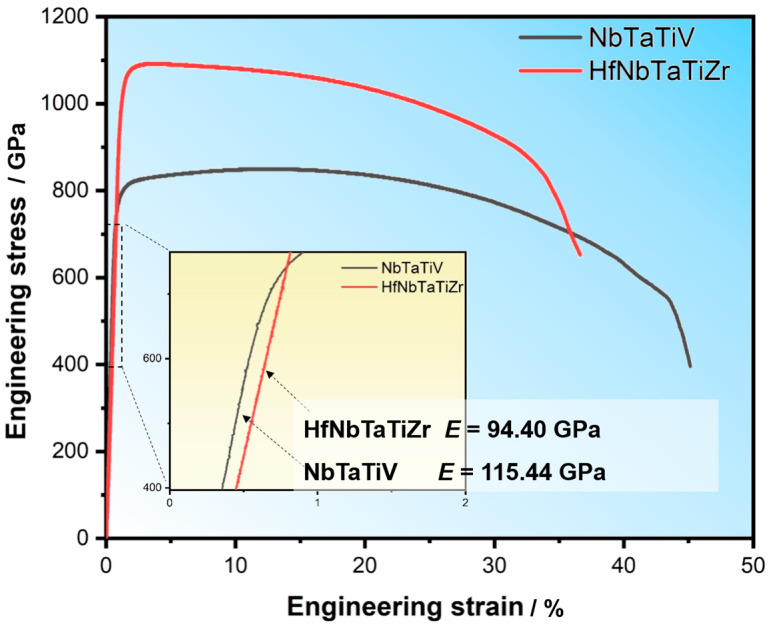
Typical tensile engineering stress–strain curves of two HEAs. The inset figure displays the enlarged parts of the curves in the elastic stage. The values of Young’s modulus (*E*) shown in the figure are the measured values for the individual typical specimens, not the average values.

The engineering stress–strain curves of NbTaTiV and HfNbTaTiZr HEAs are shown in Figure 3. Both HEAs exhibit good tensile plasticity, in contrast to many brittle refractory HEAs, such as the Al_0.5_Mo_0.5_NbTa_0.5_TiZr HEA, which broke abnormally and catastrophically under tensile loading, as described above. The uniform elongation (*ε*_u_) and total elongation (*ε*_t_) were measured to be *ε*_u_ = 12.1 ± 0.3% and *ε*_t_ = 40.1 ± 3.3% for NbTaTiV HEA, and *ε*_u_ = 2.8 ± 0.6% and *ε*_t_ = 33.6 ± 1.8% for HfNbTaTiZr HEA. Obviously, the quaternary alloy shows better plastic deformation ability than the quinary alloy.

However, the strengths of NbTaTiV are a bit lower than those of HfNbTaTiZr. The former has a yield strength (*σ*_y_) of 770 ± 16 MPa and an ultimate tensile strength (*σ*_uts_) of 835 ± 11 MPa, while for HfNbTaTiZr, *σ*_y_ = 992 ± 21 MPa, *σ*_uts_ = 1064 ± 20 MPa. The inset figure in Figure 3 shows enlarged parts of the elastic stage of the stress–strain curves. According to Hooke’s law, the values of Young’s modulus (*E*) of NbTaTiV and HfNbTaTiZr HEAs, measured using uniaxial tension tests, are 113.66 ± 6.09 GPa and 94.53 ± 1.69 GPa, respectively.

The elastic properties for the three HEAs were also measured using IEV tests. The results are listed in Table 1, in which the Young’s modulus measured by tension was also included. Comparing the *E* values for the same alloy obtained with the two different methods, it can be found that the value measured using the tension method is a little lower than that determined by IEV, namely, 3.1% lower for NbTaTiV and 0.7% lower for HfNbTaTiZr. The small difference in *E* between the two methods is consistent with the observations in steels [49] and possibly due to the loading rate effect, i.e., quasi-static loading for tension vs. dynamic loading for IEV.

In addition to Young’s modulus, the shear modulus (*G*) and Poisson’s ratio (*ν*) were also measured using IEV tests. The shear moduli of Al_0.5_Mo_0.5_NbTa_0.5_TiZr, NbTaTiV, and HfNbTaTiZr are 43.98 GPa, 42.80 GPa, and 34.98 GPa, respectively, and the Poisson’s ratios are 0.352, 0.371, and 0.362, respectively. All the measured values of elastic properties are listed in Table 1.

### 4.2. Prediction of Elastic Modulus from Composition

To further study the elastic properties of HEAs, we collected a lot of modulus data of about 130 HEAs with different compositions from published papers (e.g., [50,51,52,53,54,55,56,57,58,59,60]), including both experimentally measured data and computational simulation values. It is important to note that for Young’s modulus measured by tension or compression, to guarantee the validity of measurement, only data measured in the studies where the strains were accurately determined using an extensometer or a strain gauge were collected.

**Figure 4:**
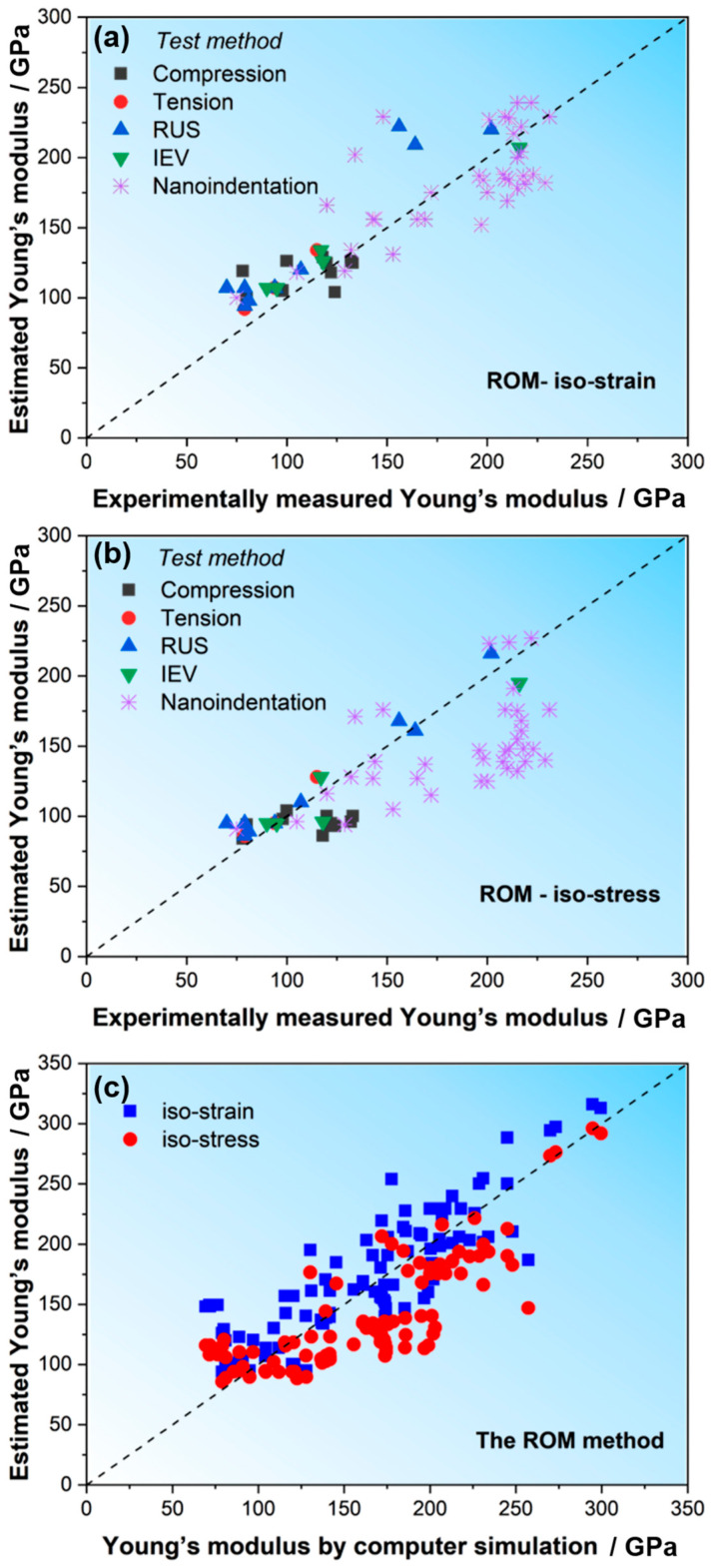
*E* estimated from ROM of HEAs versus experimental and computational simulations: (**a**) *E*_strain_ vs. *E*_exp_, (**b**) *E*_stress_ vs. *E*_exp_, (**c**) *E*_com_ vs. *E*_ROM_.

Based on the compositions of the studied HEAs in this manuscript as well as those in the literature, we estimated the E values for the two assumptions (i.e., *E*_stress_ for iso-stress and *E*_strain_ for iso-strain) according to the ROM of Equations (2) and (3). The specific values are listed in Table 2 and Table 3. To more vividly observe the predictions, we also plotted the estimated *E* values vs. experimentally measured *E* values (*E*_exp_) in Figure 4a,b and the estimated *E* vs. computer-simulated values (*E*_com_) in Figure 4c. It can be found that the Young’s modulus values of HEAs measured using various test methods are within the range of the estimated values of the ROM and are closer to the lower limits estimated by the assumption of iso-stress (*E*_stress_). However, for the computer-simulated E values, the estimation based on the assumption of iso-strain (*E*_strain_) seems to be more proper to predict the Ecom, i.e., the Ecom is closer to the upper limit.

The difference between experiments and simulations may originate from several factors. While defects in material microstructures are a major cause, other factors, such as microstructural heterogeneities and stresses induced by the solidification process, can also significantly influence the observed properties. In simulations, the material is often assumed to be defect-free or to have a controlled microstructure, leading to a closer match with the upper bound of elastic properties. However, real materials may exhibit variations in microstructure and stresses, which can affect the measured properties, particularly in localized tests such as nanoindentation. Nanoindentation, due to its localized nature, is especially sensitive to such microstructural variations and may show different results compared to bulk tests like uniaxial tension or IEV tests. This can also be illustrated by the *E* values measured by nanoindentation, which are closer to the upper limit of *E*_strain_, as shown in Figure 4a. This is in contrast to the *E* values measured using tension and IEV tests, where large specimens were usually used. As discussed above, during the nanoindentation, the volume of the materials deformed is very small and could be much smaller than the size of a grain; hence, the results of the nanoindentation are more sensitive to the local microstructure under the indenter and are less likely to be affected by defects, leading to scatter and higher values than the data obtained using other methods.

Since there is a discrepancy between the *E* values estimated using Equations (2) and (3) and the experimental or simulation data, we took the averages of the above two assumptions and calculated the average value of ROM using the following equation:

(11) $$\begin{array}{c} \bar{P}=\frac{1}{2}\left(\sum \frac{P^{i}V_{m}^{i}f^{i}}{V_{m}}+\frac{1}{\sum \frac{V_{m}^{i}f^{i}}{P^{i}V_{m}}}\right) \end{array}$$

**Figure 5:**
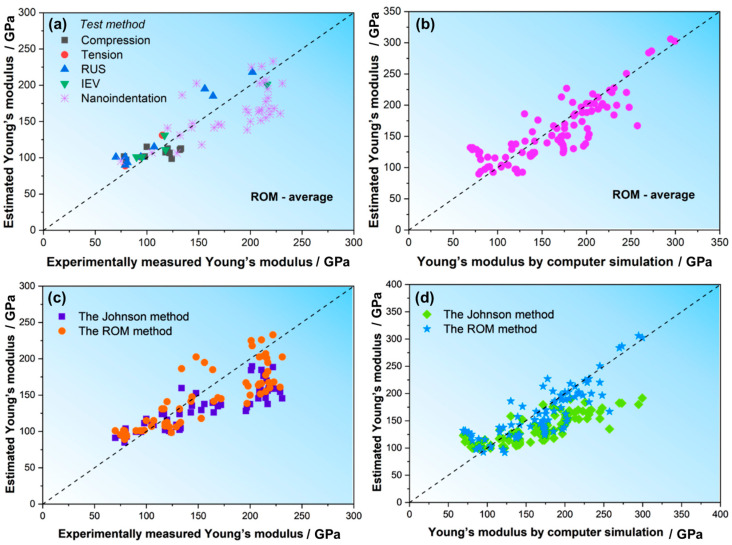
(**a**,**b**) Comparison between the *E* estimated by the average value of ROM and the experimental and simulated values. (**c**,**d**) Comparison of the experimental *E* values and computer-simulated E values with the *E* values estimated using the Johnson method and the ROM method.

The comparisons between the calculated average modulus values (*E*_avg_) according to Equation (11) and the experimental data or the simulation modulus vales are presented in Figure 5a,b. Apparently, the average modulus value of ROM can better estimate the Young’s modulus value of HEAs for both experiments and simulations.

Furthermore, we calculated the Young’s modulus of the above ~130 HEAs using the Johnson method (Equation (4)) and compared it with the experimental and calculated *E* values in the literature. The results are shown in Figure 5c,d. For comparison, the predicted *E* calculated using the above ROM method is also plotted. Apparently, the Johnson method predicts the *E* values well when the *E* is lower than ~150 GPa, while for HEAs with higher *E*, the predicted *E* values are obviously smaller than the experimental or simulated values. In contrast, the prediction of *E*_avg_ using the ROM method is better.

### 4.3. Experimental Verification of the Strategy

The results above demonstrate the effectiveness of the ROM method for predicting the Young’s modulus for the composition design of HEAs, which facilitates the application of the present composition design strategy discussed in Section 2. To further verify this strategy experimentally, we designed new alloys using this method and measured the properties of the designed alloys using experiments. NbMoTaVW alloy has excellent high-temperature strength, especially above 1000 °C, but it is prone to brittle fracture and has less than 5% compressive plasticity at room temperature [40]. In order to improve the mechanical properties, we selected this alloy system and used the composition design strategy to achieve property optimization.

**Figure 6:**
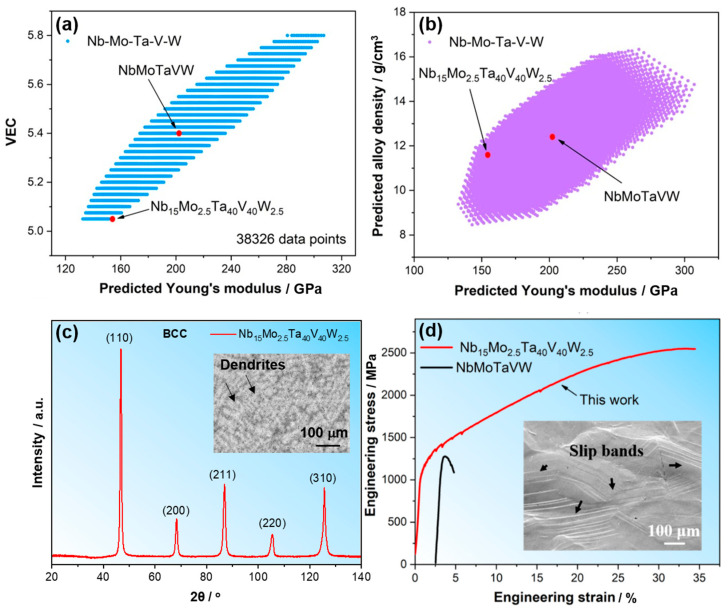
(**a**,**b**) Predicted property maps for Nb-Mo-Ta-V-W HEA system: the predicted VEC vs. the predicted Young’s modulus (**a**) and the predicted density vs. the predicted Young’s modulus (**b**). (**c**,**d**) Experimental results of the newly designed Nb_15_Mo_2.5_Ta_40_V_40_W_2.5_ refractory HEA, including the XRD profile (**c**), the BSE image of microstructure (inset of (**c**)), the typical compressive engineering stress–strain curves (**d**) of Nb_15_Mo_2.5_Ta_40_V_40_W_2.5_ and NbMoTaVW [40] HEAs, and the SEM image on the surface of a deformed specimen of Nb_15_Mo_2.5_Ta_40_V_40_W_2.5_ (inset of (**d**)).

We adjusted the composition of the Nb-Mo-Ta-V-W system by varying the atomic concentrations of each element in the range from 2.5 to 40 at.% with a step size of 2.5%. We then calculated the VEC, density, and E according to Equations (6)–(8), respectively. A total of 38,326 compositions were calculated, and the maps of predicted properties can finally be plotted, as shown in Figure 6a,b. The predicted variation trend for *E* against VEC is a trade-off, which is consistent with the strength–ductility trade-off for metallic materials [96]. The goal for this composition design is to enhance the intrinsic ductility but reduce the loss of strength or elastic modulus. Thus, we selected the alloy composition with the smallest VEC and also a relatively high *E*. According to Figure 6a, a new HEA with a composition of Nb_15_Mo_2.5_Ta_40_V_40_W_2.5_ (at.%) was finally designed. The further property map in Figure 6b illustrates that the Nb_15_Mo_2.5_Ta_40_V_40_W_2.5_ alloy is expected to have a lower density than the equimolar NbMoTaVW HEA. We then experimentally prepared the alloy with the composition of Nb_15_Mo_2.5_Ta_40_V_40_W_2.5_ (at.%) using the electric arc melting method, characterized the microstructure using XRD and SEM, and measured the mechanical properties using uniaxial compression tests.

The experimental results are shown in Figure 6c,d. The XRD profile suggests that the as-cast Nb_15_Mo_2.5_Ta_40_V_40_W_2.5_ alloy is a single-phase BCC structure. The BSE image inset of Figure 6c reveals a dendritic microstructure in this alloy, which is the typical microstructure feature of the as-cast alloy in BCC HEA and consistent with other ones [5,17,40]. The grain size for the new alloy is measured to be ~240 μm, which is a little larger than the equimolar NbMoTaVW alloy (~200 μm) prepared with a similar casting method [40]. The compressive engineering stress–strain curves of both the present new alloy and the equimolar NbMoTaVW alloy in the literature [40] are displayed in Figure 6d for comparison. Accordingly, the yield strength of Nb_15_Mo_2.5_Ta_40_V_40_W_2.5_ is 1115 ± 18 MPa, slightly lower than that of the as-cast equimolar NbMoTaVW alloy (~1246 MPa), which is consistent with the predicted decreasing trend of elastic modulus. However, the compressive fracture strain (plasticity) of the new Nb_15_Mo_2.5_Ta_40_V_40_W_2.5_ alloy is greatly improved to 35.7 ± 0.9%, much higher than ~2% for NbMoTaVW HEA. This demonstrates that by reducing VEC, the intrinsic shear plastic deformation ability is effectively enhanced. In addition, the initiation of brittle cracking is severely delayed, and many slip lines can be observed on the specimen surface (see the inset figure in Figure 6d). The dislocation slip that dominates the work-hardening ability of the material is fully developed, and a very high compressive strength of 2550 ± 75 MPa was observed. These results indicate that new compositions of HEAs with better properties can be effectively designed using the present composition design strategy.

To further confirm the promising properties, the Vickers hardness and nanoindentation tests were performed for the new HEA. The results suggest that the Vickers hardness is 4.13 ± 0.09 GPa and the nanohardness is 8.90 ± 0.91 GPa. The difference in hardness values between Vickers hardness and nanohardness originates from the indentation size effect [97]. The volume beneath the indenter is much smaller in nanoindentation than that in Vickers hardness, which significantly limits the dislocation activity and thus increases the difficulty for deformation. Nevertheless, the high hardness values suggest a high resistance to plastic deformation in the present new HEA. Moreover, it is found that the ratio between Vickers hardness and the compressive yield strength for the present HEA is 3.65, which is much higher than 3, usually for brittle BCC HEA, but agrees with ductile HEAs [98], indirectly implying the ductile nature of the present new alloy.

### 4.4. Limitations and Further Discussion

The success of the current strategy for designing new RHEAs with high ductility was verified by experiments. Thereafter, new RHEA compositions with desired properties, such as high elastic modulus, high ductility, and low densities, can thus be discovered from the property map. However, it should be noted that the present composition design strategy has limitations.

First, the yield strength of RHEAs has not yet been predicted or designed. The present strategy estimates the elastic modulus of the HEAs with the ROM and relates the Young’s modulus to the ideal tensile strength, which provides a ceiling of strength for perfect materials, but is far from the strength of real materials. A recent study by Maresca and Curtin et al. [99,100,101] provided a solution for predicting the yield strength of BCC HEAs. The calculation was based on edge dislocation strengthening, and yield strength at finite temperature (T) and finite strain rate ($\dot{\varepsilon }$) are given below:

(12) $$\begin{array}{c} \tau_{y}\left(T,\dot{\varepsilon }\right)=\tau_{y0}exp\left[-\frac{1}{0.55}{\left(\frac{k_{B}T}{{∆E}_{b}\ln\left(\frac{{\dot{\varepsilon }}_{0}}{\dot{\varepsilon }}\right)}\right)}^{0.91}\right] \end{array}$$

where $\tau_{y0}$ is the zero temperature strength; ${∆E}_{b}$ is the zero temperature energy barrier; ${\dot{\varepsilon }}_{0}$ = 10^4^ s^−1^ is a reference strain rate; $k_{B}$ is the Boltzmann constant. $\tau_{y0}$ and ${∆E}_{b}$ can be expressed as follows:

(13) $$\begin{array}{c} \tau_{y0}=0.04\alpha^{-1/3}G{\left(\frac{1+\nu }{1-\nu }\right)}^{4/3}{\left[\frac{\sum_{n}c_{n}{∆V}_{n}^{2}}{b^{6}}\right]}^{2/3} \end{array}$$

(14) $$\begin{array}{c} {∆E}_{b}=2\alpha^{1/3}Gb^{3}{\left(\frac{1+\nu }{1-\nu }\right)}^{2/3}{\left[\frac{\sum_{n}c_{n}{∆V}_{n}^{2}}{b^{6}}\right]}^{1/3} \end{array}$$

Here, *α* = 1/12 is a line-tension coefficient, *G* and ν are the shear modulus and Poisson’s ratio of the alloy, b is the Burgers vector of edge dislocation, *c_n_* is the atomic concentration for the nth element, and $∆V_{n}$ is the elemental misfit volumes. This approach has been proven to be effective for predicting the yield strengths of BCC HEAs. By combining Equation (12) with the present composition design strategy, it is expected to achieve the ability to design RHEAs with high strength and ductility. However, correctly estimating the values of parameters in Equations (12)–(14) and the scope of application of these calculations requires further investigations.

Second, the effect of microstructure on mechanical properties has not been considered in the present strategy. Actually, the microstructures of crystalline metallic materials, including grain size, phase structures, etc., have significant impacts on strength and ductility. This limits the direct prediction of mechanical properties, which is one of the challenges in this field. To include the microstructure effect, the quantitative relations of microstructure vs. properties and processing conditions vs. microstructure formation should be determined and included in the material design strategy. This requires long-term work and is out of the scope of the present study.

Although there are some limitations, the present strategy provides good estimates on the elastic modulus and VEC of RHEAs and suggests the usage of a property map to find optimal compositions for desired properties. Thus, a direction for discovering light, strong, and intrinsically ductile RHEA compositions that could deform plastically rather than fracture in a brittle manner could be indicated by the present strategy. It is expected that by combining the present strategy with additional theoretical efforts (e.g., thermo-kinetics predictions of microstructures [12,13] and quantitative predictions of mechanical properties [99]) and computational studies (e.g., machine learning [29]), more efficient composition optimization and design of RHEAs will be achieved.

## 5. Conclusions

In this manuscript, a composition design method for HEAs is proposed. The following conclusions can be drawn:

(1) The Young’s modulus of the NbTaTiV and HfNbTaTiZr HEAs was measured by both the tensile test and IEV test. No significant differences were found between the two test methods. The shear modulus and Poisson’s ratio were also measured with the IEV test for these two alloys and the Al_0.5_Mo_0.5_NbTa_0.5_TiZr HEA.
(2) According to the ROM, the elastic modulus values of 132 HEAs were estimated based on their compositions. The estimated elastic modulus was compared with the experimental or simulated values in literature and this study, and the results indicate that the average values of the ROM can estimate the elastic modulus of HEAs.
(3) A composition design strategy is proposed based on the correlations between compositions and properties, including Young’s modulus, VEC, density, and atomic size difference. Property maps can be predicted and plotted, and the optimal composition with desired properties can be reached.
(4) According to the composition design strategy, a new HEA with the composition of Nb_15_Mo_2.5_Ta_40_V_40_W_2.5_ was designed. Further tests of the as-cast new alloy, including mechanical property measurement and structure characterization, suggest that this HEA has a BCC single phase and much higher compressive strength (2550 ± 75 MPa) and plasticity (35.7 ± 0.9%) than the equimolar NbMoTaVW alloy. The experimental results validate the rationality of the design strategy.

This study justified the applicability of the ROM for predicting the elastic modulus of HEAs and further proposed a composition design strategy to estimate alloy properties directly from composition. This approach provides a practical framework for the design of HEAs with tailored mechanical properties. Nonetheless, the underlying physical mechanisms governing the relationships between composition, elastic modulus, and ductility are not yet fully understood, and further investigations are needed. Future work could focus on exploring these mechanisms through combined computational modeling and experimental studies, as well as extending the design strategy to other HEA systems. Moreover, the methodology and findings presented here can be potentially applied to the development of high-performance alloys for real engineering applications, such as structural components in extreme environments, where both high strength and sufficient plasticity are required.

## Figures and Tables

**Table 1:** Elastic properties of the studied three HEAs experimentally measured using uniaxial tension and IEV tests.

Alloy Composition	Young’s Modulus, *E* (GPa)		Shear Modulus, *G* (GPa)	Poisson’s Ratio, *ν*
	By Tension	By IEV	By IEV	By IEV
Al_0.5_Mo_0.5_NbTa_0.5_TiZr	-	118.39	43.98	0.352
NbTaTiV	113.66 ± 6.09	117.31	42.80	0.371
HfNbTaTiZr	94.53 ± 1.69	95.27	34.98	0.362

**Table 2:** Young’s modulus (*E*), shear modulus (*G*), and bulk modulus (*B*) of various HEAs experimentally measured using static tests and dynamic tests in references, as well as the estimated *E* values according to Equations (2) and (3).

Alloy	Experimental Method	Measured *E* Values (GPa)	Estimated *E* Values (GPa)		*G* (GPa)	*B* (GPa)	Ref.
			Iso-Strain	Iso-Stress			
Al_0.4_Hf_0.6_NbTaTiZr	Compression	78	119	84			[61]
Al_0.25_NbTaTiZr		118	129	86			[50]
AlNbTa_0.5_TiZr_0.5_		124	104	93			[50]
AlMo_0.5_NbTa_0.5_TiZr		122	118	95			[50]
AlMo_0.5_NbTa_0.5_TiZr_0.5_		133	125	100			[50]
Al_0.5_Mo_0.5_NbTa_0.5_TiZr		132	126	96			[50]
CrNbTiZr		120	125	100			[52]
CrNbTiVZr		100	126	104			[52]
NbTiVZr		80	100	94			[52]
NbTiV_2_Zr		98	105	98			[52]
Hf_27.5_Nb_5_Ta_5_Ti_35_Zr_27.5_	Tension	79	92	86			[62]
NbTaTiV		115	134	128			This study
HfNbTaTiZr		94	107	95			This study
CoCrFeMnNi	Resonant ultrasound spectroscopy	202	220	216	80	143	[63]
Fe_10_Nb_30_Ta_10_Ti_40_Zr_10_		107	120	110			[58]
Hf_25_Nb_12.5_Ta_12.5_Ti_25_Zr_25_		81	98	89			[58]
HfNbTaTiZr		94	107	95			[58]
HfNbTaTiZr		79	107	95		135	[64]
HfNbTiZr		70	107	95	25	106	[65]
MoNbTaTiW		156	222	168	59	139	[66]
MoNbTaTiVW		164	209	161	62	150	[66]
Nb_5_Ta_5_Ti_45_Zr_45_		79	94	86			[58]
Co_1.5_CrFeNi_1.5_Ti_0.5_	Impulse Excitation of Vibration	216	207	195			[67]
HfNbTaTiZr		90	107	95	36	62	[68]
Al_0.5_Mo_0.5_NbTa_0.5_TiZr		118	126	96	44		This study
NbTaTiV		117	134	128	43		This study
HfNbTaTiZr		95	107	95	35		This study
AlCoCuFeNiVZr	Nanoindentation	153	131	105			[69]
Al_19_Co_23_Cr_3_Fe_9_Ni_26_Ti_20_		197	152	125			[53]
AlCo_13_Cr_46_Fe_34_Ni_5_Ti		222	239	227			[53]
Al_13_Co_18_Cr_21_Fe_19_Ni_18_Ti_11_		211	184	148			[53]
AlCoCrFeNiTi_0.1_		229	182	140			[54]
AlCoCrFeNi_2_Ti_0.1_		209	185	146			[54]
AlCoCrFeNiTi_0.1_Si_0.1_		215	178	132			[54]
AlCoCrFeNi_2_Ti_0.1_Si_0.1_		219	181	139			[54]
AlCoCuFeNi		143	156	127			[70]
AlCoCuFeNi		165	156	127			[70]
AlCoCrFeNi		198	184	141			[54]
AlCoCrFeNi_2_		196	187	147			[54]
Al_0.2_Co_1.5_CrFeNi_1.5_Ti		210	169	134			[71]
AlCrTiVZr		129	119	94			[69]
AlCrFe_2_Ni_2_		218	187	148			[56]
AlCrFe_2_Ni_2_(MoNb) _0.1_		223	188	148			[56]
AlCrMnMoNiZr		172	175	115			[69]
Al_0.5_CrMoNbV		208	188	139	74		[72]
AlCrMoNbV		200	175	125	71		[72]
Al_0.1_CrMoNbV		215	200	155	77		[73]
Al_0.56_Mo_1.18_Nb_1.19_Ta_1.15_VW_1.28_		231	229	176			[74]
CrCuNbNiTi		169	156	137			[69]
(CoCrFeNi)_5.5_Cr_0.5_		201	227	223			[75]
(CoCrFeNi)_5_Cr		211	228	224			[75]
(CoCrFeNi) _4_CrAl		213	217	191			[75]
CrHfTiVZr		105	118	96			[69]
CrMoNbTiW		215	239	175	85		[76]
CrMoNbV		217	204	161	78		[72]
Cr5NbTaTiV		134	202	171			[77]
CrNbTaTiV		144	156	139			[77]
Hf_15_Nb_20_Ta_10_Ti_30_Zr_25_		75	100	91	29		[78]
MoNbTaVW		148	229	176			[79]
MoNbTaVW		209	229	176			[74]
MoNbTaTiW		217	222	168	82		[80]
NbTaTiWZr		120	166	116			[69]
NbTaTiV		132	134	128			[77]

**Table 3:** Elastic modulus by computer simulation of various HEAs in the literature, as well as the estimated *E* according to Equations (2) and (3).

Alloy	Computer Simulated *E* Values (GPa)	Estimated *E* Values (GPa)		*B* (GPa)	*G* (GPa)	*ν*	Ref.
		Iso-Strain	Iso-Stress				
Al_0.25_MoNbTiV	168.0	160.5	128.5	161.4	63.3	0.326	[51]
Al_0.4_MoNbTiV	171.1	156.4	123.8	159.7	64.7	0.322	[51]
Al_0.45_MoNbTiV	171.7	157.4	124.9	159.1	65.0	0.320	[51]
Al_0.5_MoNbTiV	172.1	155.4	122.7	158.6	65.2	0.319	[51]
Al_0.6_MoNbTiV	173.3	153.5	120.7	157.5	65.8	0.317	[51]
Al_0.75_MoNbTiV	173.9	151.5	117.4	155.9	66.2	0.314	[51]
AlMoNbTiV	185.4	146.7	114.0	165.9	70.6	0.314	[51]
AlMoNbTiV	174.4	146.7	114.0	153.6	66.5	0.311	[81]
Al_1.25_MoNbTiV	174.4	143.0	110.6	151.4	66.7	0.308	[51]
Al_1.5_MoNbTiV	173.8	140.2	107.3	147.4	66.7	0.303	[82]
AlMoNbV	196.7	155.2	113.4	174.2	75.0	0.310	[83]
AlNbTiVZr	122.6	94.6	88.4	-	-	-	[84]
AlNiCoCu	116.0	142.7	115.7	41.0	56.0	0.030	[85]
AlNiCoCrFeTi	161.0	169.2	134.3	154.0	61.0	0.330	[85]
Al_0.3_CoCrFeNi	248.0	210.4	182.8	196.0	96.0	0.289	[59]
Al_0.5_CoCrFeNi	231.0	201.8	166.3	190.0	89.0	0.297	[59]
AlCoCrFeNi	201.0	184.2	140.6	183.0	76.0	0.317	[59]
Al_1.3_CoCrFeNi	203.0	175.8	130.8	170.0	78.0	0.301	[59]
Al_1.5_CoCrFeNi	202.0	170.8	125.7	167.0	78.0	0.297	[59]
Al_2_CoCrFeNi	199.0	160.2	116.0	159.0	77.0	0.291	[59]
CoCrFeNi	226.0	225.7	221.7	-	85.0	-	[86]
CoCrCuFeNi	217.0	206.1	193.7	-	71.0	-	[86]
CoCrCuFeNi	234.0	206.1	193.7	179.0	91.0	0.282	[60]
CoCrCuFeNiTi	145.4	184.9	167.4	157.0	54.0	0.346	[60]
CoCrCuFeNiTi_0.1_	223.1	203.4	189.9	175.0	87.0	0.288	[60]
CoCrCuFeNiTi_0.2_	213.1	200.9	186.5	173.0	82.0	0.294	[60]
CoCrCuFeNiTi_0.3_	205.9	198.5	183.4	172.0	80.0	0.298	[60]
CoCrCuFeNiTi_0.4_	200.4	196.2	180.5	171.0	78.0	0.303	[60]
CoCrCuFeNiTi_0.5_	187.1	194.1	177.9	169.0	71.0	0.313	[60]
CoCrFeNiTi	130.3	195.1	176.8	175.0	47.0	0.376	[60]
CoCrFeMnNi	207.0	219.9	216.3	-	-	0.313	[87]
CoCrFeNi(Al_0.3_Ti_0.2_)_0.25_	171.9	219.6	206.6	-	-	-	[88]
CoCrFeNi(Al_0.3_Ti_0.2_)_0.5_	184.5	214.1	194.5	-	-	-	[88]
CoCrFeNi(Al_0.3_Ti_0.2_)_0.75_	194.1	209.0	184.5	-	-	-	[88]
CoCrFeNi(Al_0.3_Ti_0.2_)	205.6	204.3	176.2	-	-	-	[88]
CrFeMoNiW	273.2	297.2	276.2	-	105.2	0.298	[89]
CrFe_1.2_MoNiW	270.0	294.2	273.3	-	103.9	0.299	[89]
CrHfNbTiZr	104.4	113.8	93.7	117.2	38.6	0.352	[90]
CuIrNiPdPtRh	213.0	239.8	185.5	-	84.0	-	[86]
CrMoNbTaW	231.0	254.5	199.9	232.0	86.0	0.334	[91]
CrMoNbTaTiVZr	130.9	161.4	123.1	166.3	49.7	0.369	[81]
CrMoNbTaTiVZr	141.8	161.4	123.1	163.9	52.3	0.356	[51]
CrMoNbTaTiVWZr	166.7	190.9	134.2	179.0	63.3	0.345	[81]
CrMoNbTaTiVWZr	175.3	190.9	134.2	176.6	65.7	0.335	[51]
CrMoTiV	195.2	207.9	168.3	193.4	73.3	0.330	[83]
CrMoMnW	299.5	312.8	291.9	-	119.4	0.255	[89]
CrMoNiW	294.7	315.9	296.1	-	112.3	0.312	[89]
HfMoNbTaTiZr	136.6	137.0	104.8	160.8	50.3	0.358	[51]
HfMoNbTaW	195.0	207.4	140.2	192.0	74.0	0.330	[91]
HfNbTaTiV	97.0	120.3	110.3	135.0	35.0	0.380	[92]
HfNbTaTiZr	104.1	107.0	94.7	136.3	37.9	0.373	[51]
HfNbTaTiZr	88.9	107.0	94.7	140.1	31.9	0.394	[51]
HfNbTiVZr	95.0	95.0	89.7	126.6	34.6	0.375	[51]
Mo_0.8_NbTiV_0.2_Zr	137.6	134.4	102.2	132.9	51.8	0.327	[51]
Mo_0.8_NbTiV_0.5_Zr	137.9	134.0	103.3	134.6	51.9	0.329	[51]
MoNbTiV_0.25_Zr	141.6	142.1	105.3	137.4	53.3	0.328	[51]
MoNbTiV_0.5_Zr	141.7	141.5	106.1	137.6	53.3	0.328	[51]
MoNbTiV_0.75_Zr	141.5	140.9	106.8	138.0	53.3	0.329	[51]
MoNbTiVZr	141.1	140.4	107.5	138.5	53.2	0.330	[81]
MoNbTiV_1.25_Zr	141.4	139.9	108.2	140.6	53.1	0.332	[51]
MoNbTiV_1.5_Zr	141.6	139.5	108.8	141.2	53.1	0.334	[51]
Mo_0.8_NbTiZr	137.2	134.6	101.4	132.2	51.7	0.327	[51]
Mo_0.9_NbTiZr	139.5	138.8	102.9	134.4	52.6	0.327	[51]
MoNbTaTiV	139.2	170.6	144.2	181.2	50.7	0.372	[51]
MoNbTaTiVZr	71.9	148.2	115.8	152.6	26.3	0.421	[81]
MoNbTaTiVZr	69.4	148.2	115.8	156.6	24.3	0.426	[51]
MoNbTaVW	204.5	229.4	175.8	245.1	75.1	0.361	[51]
MoNbTaVW	200.0	229.4	175.8	222.0	82.0	0.348	[93]
MoNbTaWZr	163.0	203.5	130.3	160.0	61.0	0.330	[91]
MoNbTaW	228.7	250.2	190.3	261.6	84.4	0.354	[51]
MoNbTiV	161.1	166.3	135.7	164.5	60.3	0.337	[51]
MoNbTiV	178.6	166.3	135.7	165.7	67.6	0.320	[81]
MoNbTiV	173.5	166.3	135.7	172.6	65.1	0.330	[83]
MoNbTiVZr	139.5	140.4	107.5	138.7	52.5	0.333	[51]
MoNbTiVZr	127.8	140.4	107.5	144.9	47.2	0.353	[51]
MoNbTiZr	140.1	142.7	104.4	136.6	52.7	0.329	[51]
MoNbTiZr	141.7	142.7	104.4	137.3	53.3	0.328	[51]
MoNbTaW	245.0	250.2	190.3	236.0	92.0	0.327	[94]
MoNbTaTiW	196.0	222.3	168.0	200.0	73.0	0.337	[94]
MoNbTaVW	218.0	229.4	175.8	234.0	81.1	0.345	[51]
MoNbReTaW	245.0	288.3	212.8	257.0	91.0	0.341	[91]
MoTiVZr	76.4	149.5	108.2	139.0	27.1	0.408	[51]
MoTiVZr	71.6	149.5	108.2	141.4	26.1	0.415	[81]
MoTiWZr	185.9	211.0	124.6	-	-	-	[57]
MoTaTiW	177.7	253.8	200.2	-	-	-	[57]
MoTaTiZr	155.7	162.3	116.7	-	-	-	[57]
MoTaTiWZr	175.5	206.0	133.4	-	-	-	[57]
MoTaTiVZr	120.5	156.9	118.3	156.0	43.9	0.371	[51]
MoTaTiVZr	115.5	156.9	118.3	154.3	43.4	0.375	[81]
MoTaWZr	185.6	227.9	138.5	-	-	-	[57]
NbTiVZr	121.1	100.2	94.0	118.6	45.7	0.330	[81]
NbTiVZr	85.6	100.2	94.0	133.1	30.7	-	[95]
NbTiVZr	119.7	100.2	94.0	117.9	45.1	0.331	[51]
NbTiV_2_Zr	91.0	104.7	98.2	142.6	32.7	-	[95]
NbTaTiWV	257.3	186.8	147.0	198.6	100.1	0.285	[51]
Nb_10_Ta_25_Ti_60_Zr_5_	80.0	129.3	120.6	131.0	29.0	-	[55]
Nb_10_Ta_25_Ti_55_TZr_10_	79.0	126.0	115.2	129.0	28.0	-	[55]
Nb_10_Ta_25_Ti_50_Zr_15_	89.0	122.9	110.4	126.0	32.0	-	[55]
Nb_10_Ta_25_Ti_45_Zr_20_	81.0	119.9	106.1	124.0	29.0	-	[55]
TaTiWZr	171.3	180.5	118.8	-	-	-	[57]

## Data Availability

The original contributions presented in this study are included in the article. Further inquiries can be directed to the corresponding authors.

## Abbreviations

The following abbreviations are used in this manuscript:

HEAs	High-entropy alloys.
MPEAs	Multiple-principal element alloys.
CALPHAD	Calculation of phase diagram.
BCC	Body-centered cubic.
VEC	Valence electron concentration.
RHEAs	Refractory high-entropy alloys.
IEV	Impulse excitation of vibration.
ROM	Rule of mixture.
DOS	Density of states.
XRD	X-ray diffraction.
BSE	Backscattered electron.
SEM	Scanning electron microscope.
Eavg	Average modulus.
